# Emotional reactivity to appraisals in patients with a borderline personality disorder: a daily life study

**DOI:** 10.1186/s40479-018-0095-7

**Published:** 2018-11-13

**Authors:** Marlies Houben, Laurence Claes, Ellen Sleuwaegen, Ann Berens, Kristof Vansteelandt

**Affiliations:** 1 Faculty of Psychology and Educational Sciences, KU Leuven, Tiensestraat 102, Box 3713, 3000 Leuven, Belgium; 2 Faculty of Medicine and Health Sciences, University of Antwerp, Universiteitsplein 1, 2610 Wilrijk, Belgium; 3 University Department of Psychiatry, Campus Psychiatric Hospital Duffel, Stationsstraat 22c, 2570 Duffel, Belgium; 4 KU Leuven, University Psychiatric Center KU Leuven, Leuvensesteenweg 517, 3070 Kortenberg, Belgium

**Keywords:** Borderline personality disorder, Emotional reactivity, Emotional appraisals, Trust and disappointment in self and others, Daily life

## Abstract

**Background:**

Emotional instability, consisting of patterns of strong emotional changes over time, has consistently been demonstrated in daily life of patients with a borderline personality disorder (BPD). Yet, little empirical work has examined emotional changes that occur specifically in response to emotional triggers in daily life, so-called emotional reactivity. The goal of this study was to examine emotional reactivity in response to general emotional appraisals (i.e. goal congruence or valence, goal relevance or importance, and emotion-focused coping potential) and BPD-specific evaluations (trust and disappointment in self and others) in daily life of inpatients with BPD.

**Methods:**

Thirty inpatients with BPD and 28 healthy controls participated in an experience sampling study and repeatedly rated the intensity of their current emotions, emotional appraisals, and evaluations of trust and disappointment in self and others.

**Results:**

Results showed that the BPD group exhibited stronger emotional reactivity in terms of negative affect than healthy controls, however only in response to disappointment in someone else. BPD patients also showed weaker reactivity in positive affect in response to the appraised importance of a situation; the more a situation was appraised as important, the higher the subsequent positive affect for healthy controls only, not the patient group.

**Conclusions:**

These findings show that appraisals can trigger strong emotional reactions in BPD patients, and suggest that altered emotional reactivity might be a potential underlying process of emotional instability in the daily life.

## Background

The ways in which our emotions change over time are indicative of our psychological well-being and closely linked to psychopathology such as borderline personality disorder (BPD) [1]. Indeed, BPD is a disorder that has emotion dysregulation at its core [2]. As such, BPD has been linked to the experience of unstable and changeable emotions in daily life [3–6]. Still, most studies have examined overall patterns of emotional changes. Relatively little research so far has investigated emotional changes in response to contextual information, such as appraisals and evaluations of the (social) environment, providing limited insight into possible processes underlying emotional changes over time. In this study, we examined the role of general emotional appraisals and BPD-specific evaluations, that is, the appraised trust and disappointment in self and others. This was done by examining emotional reactivity in daily life in response to different emotional appraisals related to goal congruence, goal relevance and emotion-focused coping potential, and to BPD-specific evaluations concerning the experience of trust and disappointment in self and others.

### Borderline personality disorder and emotional instability

Borderline personality disorder (BPD) is a pervasive and debilitating disorder that is characterized by severe affective dysregulation [2, 7]. This has not only been detected in lab studies and studies using trait questionnaires (see an overview by Carpenter and Trull [8]) but also been supported by numerous daily life studies that examined the emotional functioning of BPD patients in an ecologically valid way. Typically, these daily life studies use experience sampling methods, in which participants repeatedly report their emotional states in daily life, allowing researchers to track the ups and downs of emotional states of participants in their own natural environment. Such studies have consistently shown that BPD patients are characterized by strong affective instability in daily life, which is reflected in larger fluctuations in their affective experiences over time, more abrupt changes in emotional intensity, and larger changes between positive and negative emotional states over time [1, 3–6]. Although these daily life studies elucidate the nature of emotional instability in the daily lives of those with BPD, little is known about the processes with which these unstable emotional patterns in daily life come about.

One way of obtaining a better understanding of these emotional ups and downs, and which processes potentially are driving them, is to investigate emotional change in response to situational triggers. Indeed, emotions and changes in emotional states typically occur in response to changes in the internal (i.e. thoughts, memory processes, evaluations) or external environment (i.e. events) of a person. In line with this proposition, patterns of emotional instability in persons with BPD are assumed to reflect strong reactivity to emotional stimuli in the external and internal environment [2, 7]. As a consequence, to better understand the nature of emotional instability in those with BPD, it is crucial to, not only examine overall patterns of emotional change, but also further explore which factors elicit emotional changes in daily life. In this study, we focused on two different types of emotional triggers. First, we examined emotional changes in response to general emotional appraisals, which are assumed to be general factors underlying emotions. Second, we examined emotional responses to BPD-specific evaluations related to trust and disappointment in self and others.

### The role of emotional appraisals

Some studies examining emotional reactivity in daily life using experience sampling methods have focused on the type of events or situations that people encountered, and how emotions changed accordingly. For example, daily life studies in major depressive disorder have described the so-called mood-brightening effect in response to daily positive events, showing that persons with a major depressive disorder exhibit greater reductions in negative affect in response to daily positive events than healthy controls [9–11]. However, situations or events that people encounter are rarely objectively positive or negative. Instead, in most cases, they can be evaluated or appraisals in many different ways, depending on an individual’s previous experiences, his or her concerns, well-being and coping potential [12]. As a result, emotional reactions might not necessarily be shaped by the mere occurrence of certain events, but instead by the subjective meaning given to these events. In order to better understand differences in emotional changes in daily life between persons with and without BPD, it is crucial to examine the role of evaluations or appraisals of daily life experiences. In line with this idea, BPD has often been linked to information processing biases [13], highlighting the importance of appraisals or evaluations, rather than the mere occurrence of different types of events. Moreover, BPD patients have been found to display higher levels of average negative affect and lower levels of average positive affect following high levels of appraised stress related to events or activities in daily life than psychotic patients or healthy participants, suggesting stronger reactivity to appraisals in BPD patients [14].

The idea of the importance of appraisals for emotional reactions is also advocated by the appraisal theories of emotion [15, 16]. These theories state that whenever a stimulus is presented (which could be an external stimulus such as a specific event, a person, a situation, or an internal stimulus such as a thought, memory etc.), this stimulus is evaluated or appraised in terms of several fundamental and primary variables such as the goal relevance of the stimulus (i.e., whether the stimulus is important for you), and the goal congruence (i.e., whether something is in line with your goal and thus positive, or interferes with the goal, and thereby negative). Moreover, secondary appraisals related to accountability and coping (e.g., emotion-focused coping potential: the degree to which you thinkyou can emotionally cope with it) also take place. How a stimulus is appraised in terms of these variables will determine whether, and if so, which emotion is produced, and the intensity of that emotion. As such, the way people evaluate or appraise aspects of their environment is crucial for determining their emotional state. Previous research has examined the relation between appraisals and subsequent emotional states in daily life of a general population [16]. Goal congruence (i.e., whether something is in line with your goals and thus positive, or interfering with your goals, and thus negative), other-agency (i.e., to what extent someone else is responsible), emotion focused coping (i.e., the extent to which you think you can emotionally cope) and future expectancy (i.e., the extent to which you think future events will turn out the way you want) were shown to be related to more positive emotions (and less negative emotions) at the following time point. Although such appraisals play a fundamental role in normal or typical emotion generation processes, still, individual differences exist among people concerning the relation between appraisals and emotions and concerning the strength of this relation [16]. As a consequence, it is not clear whether and how this relation is different between people suffering from BPD and people without psychopathological complaints. So far, no daily life studies have examined the relationship between BPD and emotional reactivity to these general emotional appraisals. Combining appraisal theory and BPD research holds the potential to bridge fundamental affective science and clinical science and to provide novel insights that can help to further enhance our understanding of emotional reactivity in BPD patients.

Although (daily life) studies on this topic are scarce, a surge of studies has examined emotional reactivity in the lab in response to positive, negative or neutral stimuli, which is related to the appraisal of goal congruence (i.e., whether something is appraised as positive or negative). Based on a review of existing studies [17], results are inconclusive regarding the presence of heightened reactivity, regarding the type of stimulus (positive, neutral, negative) that elicits reactivity, and regarding the response system (physiological responding, subjective experience etc.) in which altered reactivity is detected. However, one limitation is that these studies typically use standardized stimuli. More recent studies have examined reactivity in the lab in response to personally-relevant stimuli. Therefore, these findings might be more relevant for daily life studies. One study [18] used auditory stimuli and showed that individuals with BPD reported stronger negative responses to personally relevant unpleasant sounds, and weaker positive responses following standardized non-personal pleasant sounds compared to healthy controls. These findings might suggest that also in daily life, individuals with BPD might emotionally respond stronger in their negative emotions to situations that are appraised as negative, and less strong in their positive emotions in response to positive appraisals. In another lab study [19], however, no heightened reactivity was found in response to audio recordings of negative or neutral personally relevant stories in the lab.

Moreover, to our knowledge, previous studies have not examined emotional change in response to appraised emotional coping potential directly. However, BPD has been consistently linked to emotional regulation difficulties, and self-reported inability to cope with emotional experiences. This is assumed to underlie emotional instability [7, 19]. Therefore, the appraised emotion focused coping potential might be an important trigger of emotional change for those with BPD.

### The role of BPD specific evaluations

Next to general emotion appraisals, evaluations that reflect vulnerabilities related to the self and to others that have been specifically linked to BPD could be strong triggers of emotional change in those with BPD. More specifically, we investigated interpersonal and intrapersonal evaluations of trust and disappointment in self and others, which might play an important role in shaping emotional experiences, especially in BPD patients.

Not only emotional instability, also interpersonal dysfunction is central in BPD [20–22]. As such, persons with BPD are more negative in how they view others and in their expectations of others [21, 23], and are characterized by maladaptive cognitive schemas involving expectations of abuse and by mistrust in others [24, 25]. Moreover, they display mistrust in others during interpersonal interactions as shown during trust games in the lab [21, 26]. Because of the centrality of interpersonal problems [27], interpersonal evaluations are likely very potent triggers of emotional change. In line with this idea, some previous daily life studies have demonstrated the importance of interpersonal triggers for emotional change in persons with BPD. For example, in comparison to healthy controls BPD patients exhibited a greater increase in negative affect in daily life when they perceived their interaction partners as less communal, and a smaller increase in positive affect when they perceived more communal behavior in others [28]. Relatedly, individuals with BPD, compared to healthy controls reported more negative affect during interactions in which they perceived others as more cold-quarrelsome [29]. Next, BPD patients reported that increases in tension or momentary higher levels of tension in daily life were likely to be preceded by instances of rejection, being alone, and failure [30]. Moreover, in those with BPD, rejection and disagreement in daily life were stronger predictors of hostility, and rejection was a stronger predictor of sadness than in those with depression [31]. In line with the idea of rejection being a crucial trigger, BPD has been linked to rejection-rage contingency, showing that BPD patients reacted with more rage in response to perceived rejection, than healthy participants did [32]. Last, the level of BPD symptoms has been shown to moderate the relationship between the experience of momentary unstable mood and a range of different situational triggers, including being offended and disappointed [33]. To further extend these findings regarding interpersonal triggers of emotional change, we examined the importance of trust in others, since it has been linked to BPD by previous studies, and disappointment in others, since this has already been shown to be a potential trigger of emotional change.

Additionally, persons with BPD not only have negative views and interpretations of others, but are also characterized by a negative self-image. As such, BPD has been linked to maladaptive cognitive schemas in which persons with BPD view themselves as bad and inadequate [34]. Moreover, BPD has consistently been linked to low self-esteem [27, 35]. Based on these findings, we expect that, not only how others are perceived, but also how persons with BPD perceive themselves in terms of trust and disappointment, could play a prominent role in emotional change [27, 35]. However, so far, limited research has focused on emotional reactivity in response to intrapersonal evaluations.

### Reactivity in positive and negative emotions

Most daily life studies examining emotional processes in relation to BPD have mainly focused on negative emotions. However, limited research examining positive emotions does indicate that persons with BPD also experience positive emotions in daily life, although less frequently [36] compared to healthy controls. Moreover, BPD has also been linked to changes in the intensity of positive emotions in daily life, although the association with intense changes in negative emotions was strongest [1]. Because not only negative emotions, but also positive emotions are assumed to be shaped by appraisals and evaluations [15], it is important to examine differences in reactivity in both positive and negative emotions between persons with BPD and healthy controls. This research is needed in order to obtain a more comprehensive picture of emotion and emotion dysregulation in BPD.

### Current study

The goal of the current study^1^ is to obtain a better understanding of what is driving patterns of emotional change (i.e., instability) in patients with BPD. This was done by examining emotional reactivity in daily life, in response to (1) general emotional appraisals (related to goal congruence, goal relevance, and emotion-focused coping potential), that have been shown to play an important role in shaping emotional experiences in general, and (2) evaluations of trust and disappointment in self and in others, which reflect vulnerabilities that are deemed specific for those with BPD. We examined whether these appraisals and evaluations in daily life could give rise to stronger emotional reactions in BPD patients compared to healthy participants. However, this study does not (and is unable to) address the question of whether similar daily life experiences are appraised or evaluated in a different way by persons with and without BPD.

In response to the primary emotional appraisals related to goal congruence and goal relevance, we hypothesized no differences between those with and without BPD regarding the degree of reactivity, as these emotional appraisals are assumed to play a central role in typical emotion generation in the general population. Moreover, previous studies [17] have found inconsistent results regarding differences in reactivity to personally relevant stimuli in the lab. Regarding the secondary appraisal related to the emotion-focused coping potential, we hypothesized that the degree to which people feel they can emotionally cope, might be linked to stronger emotional changes in those suffering from BPD. This hypotheses was rooted in research indicating that emotional instability is linked to self-reported inability to cope with emotional experiences [7, 19].

Regarding the BPD-specific evaluations, we hypothesized that patients with BPD are more reactive to disappointment and (lack of) trust in self and others compared to healthy controls, as previous findings have indicated that persons with BPD might be especially vulnerable to these kind of evaluations [24, 25, 33, 35]. We expected heightened reactivity, mainly in negative emotions, as previous studies have shown that BPD is most strongly associated with more changeable negative emotions in daily life [1].

## Methods

### Participants

The clinical sample consisted of 30 volunteering patients^2^ that were currently admitted to a Belgian psychiatric hospital and were receiving treatment for BPD in specialized treatment units (University Psychiatric Center KU Leuven, Campus Kortenberg or psychiatric hospital Duffel). The presence of a BPD diagnosis was established by the staff during the intake procedure before entering the treatment, and was confirmed using the Assessment of DSM-IV Personality Disorders-Borderline scale (ADP-IV- Borderline scale [39]), which has shown acceptable concordance with the Structured Clinical Interview for DSM-IV-Axis II borderline personality disorder section (SCID-II - Borderline section) for the categorical diagnosis (kappa = 0.54 [40]). Based on the average dimensional score of the ADP-IV – Borderline scale (*M* = 56,83, *SD* = 7.78), this sample scored very high on BPD pathology, according to norm scores in the Flemish population [41]. Moreover, they reported high levels of depressive symptoms, also scoring above the cut-off of 9 which is indicative of a possible major depressive disorder diagnosis, according to the Psychiatric Diagnostic Screening Questionnaire—Major Depressive Disorder Scale (PDSQ-MDD Scale; *M* = 13.57, *SD* = 4.61 [42]). The average age in the patient sample was 29.03 (*SD* = 8.75). The sample was largely female (87%). Most (73%) were single, 7% was married, and 20% was divorced. For 20% of the sample, highest completed education level was primary education, 37% secondary education, and 20% tertiary education. Data was missing for 23%. Most patients were currently taking psychotropic medication (93%) such as antidepressants (73%), atypical antipsychotics (50%), typical antipsychotics (37%), and benzodiazepines (37%).

Additionally, 28 volunteering healthy control participants from the community were recruited and matched on age and gender to the patient sample. Therefore, the control sample was similar in age (*M* = 29.29, *SD* = 8.70; *t* (56) = − 0.11, *p* = .91), and the majority of participants was females (86% of the sample). Of all healthy participants, 25% was single, 18% was married, and data was missing for 57%. The highest completed education level was secondary education for 36% of the sample, and tertiary education for 57% of the sample. Education data was missing for 7%. The healthy participants were recruited from the general community by research assistants on a volunteering basis, and none of them reported mental health problems or current use of psychotropic medication using a self-reported screening questionnaire with open-ended questions about (history) of mental problems, hospitalizations and medication use. As a consequence, the healthy sample scored low both on BPD features according to the ADP-IV-Borderline scale (*M* = 19.52, *SD* = 8.19; this is within the normal range, based on what is expected in a Flemish population [41]), and depressive symptoms according to the PDSQ-MDD scale (*M* = 2.78, *SD* = 3.21, which is considerably below the cutoff of 9, indicating a possible MDD diagnosis). These scores on BPD and depressive symptoms were significantly lower than those of the patient sample (*t* (55) = 17.64, *p* < .001 for BPD symptoms; *t* (55) = 10.13, *p* <. 001 for depressive symptoms).

### Procedure

Participants were individually tested. After being informed about the study signing the informed consent form, participants completed a set of self-report questionnaires. Next, they were trained on how to use a Tungsten E palmtop to complete questionnaires, after which they participated in eight days of experience sampling (ESM [43, 44]). During these eight days, participants carried these palmtops with them in their daily lives. The devices were programmed to emit a beeping signal 10 times a day during waking hours (standard between 8.30 AM and 9.30 PM, with one beep randomly programmed in each of ten equal time intervals), announcing a short questionnaire inquiring about their current appraisals and emotions. The average time interval (in hours) between consecutive beeps was similar for BPD patients and healthy controls (*M* = 1.33, *SD* = 0.06 for BPD; *M* = 1.33, *SD* = 0.05 for healthy controls; *t* (56) = 0.002, *p* = .999), and was chosen to represent a balance between duration of sampling (i.e. multiple days) and the frequency of sampling within each day.

### Measures

#### The assessment of DSM-IV personality disorders (ADP-IV)- borderline personality disorder scale

We used the borderline personality disorder subscale of the ADP-IV [39], which is a self-report scale that consists of 10 trait items that assess the DSM-IV-TR (which are unchanged in DSM-5) diagnostic criteria for BPD, both in a categorical and dimensional manner. Each item is scored on a seven-point scale to indicate to what degree the trait applies to oneself, and an additional distress rating on a three-point scale. The trait scores provide a dimensional score for each item and can be summed to obtain a total dimensional BPD score. A categorical assessment of BPD is obtained by first counting the number of items that are scored at least 5 on the trait scale and at least 2 on the distress scale. Next, five items or more fulfilling this criterion was indicative of a BPD diagnosis. Internal consistency was good in our sample (α = 0.96).

#### Psychiatric diagnostic screening questionnaire—Major depressive disorder scale (PDSQ-MDD scale)

The PDSQ is a reliable and valid self-report questionnaire in the assessment of symptoms of several DSM–IV Axis I disorders in psychiatric patients [42]. The PDSQ-MDD Scale assesses the DSM–IV major depressive disorder diagnostic criteria, using 21 items that are scored 1 *(present)* or 0 *(absent).* A dimensional score can be obtained by counting the number of symptoms present. A sum score of 9 or more symptoms is considered indicative of a possible major depressive disorder diagnosis.

#### ESM items

At each measurement occasion, participants rated current emotional states by indicating to what degree they were currently experiencing anger, depressive feelings, anxiety, stress, happiness, and relaxed feelings, using a rating scale ranging from 0 (*not at all*) to 100 (*very much*). Based on the two positive emotions items and the four negative items, an average positive affect (PA) and average negative affect (NA) scale was constructed. Reliability estimates were obtained following suggestions by Nezlek [45], and showed good to excellent reliability for PA (estimate = .61 at the level of the measurement occasion; estimate = .99 at the person level) and for NA (estimate = .53 at the level of the measurement occasion; estimate = .99 at the person level).

Next, assessment of the general emotion appraisals (goal relevance and goal congruence, and the emotion-focused coping potential) were collected using the following questions respectively: “Think about what determines your emotions right now. To what degree is this important to you?/To what degree is this positive or negative for you?/ To what degree do you think you can cope emotionally?”, each scored on a scale ranging from 0 (*not at all/very negative*) to 100 (*very much/very positive*). These items have been adopted from a previous daily life study on appraisals [15].

Regarding the BPD-specific evaluations, participants were asked to rate to what degree they were disappointed in themselves and in someone else, each scored on a scale ranging from 0 (*not at all*) to 100 (*very much*), and to what degree they had trust in themselves and in someone else, each scored from 0 (*not at all*) to 100 (*very much*).

Considering the absence of remuneration for participation in the study, compliance to the experience sampling was fair for BPD patients, with an average compliance of 65.80% (*SD* = 19.26; Median = 66.90; 83% of participants had a compliance of at least 50%), yielding an average of 53.40 repeated assessments per person (*SD* = 15.16, range = 19–76), and good for healthy controls with an average of 84.24% compliance (*SD* = 12.13; Median = 85.45; all participants had a compliance of at least 50%), yielding an average of 67.86 repeated assessments per person (*SD* = 10.72, range = 38–91). However, compliance differed significantly between groups (t(56) = − 4.33, *p* < .01).

### Statistical analysis

We used multilevel models to analyze the data, which take the dependency of measurements (i.e., repeated measurements nested within participants) into account. All analyses were conducted in HLM7.

In the first set of analyses, we examined reactivity to all the general emotional appraisals. For PA and NA separately, we estimated a two-level model in which affect (positive or negative affect) at time *t* was predicted by a random intercept, and by each emotional appraisal at the previous time point and affect at the previous time point, using random slopes. We included the appraisals measured at the previous time point, because this is the only way to assure that the predictor occurred *before* the emotional response. A similar approach has been used in previous studies [15]. All predictors were centered within-person to control for the effect of possible differences between participants (and groups) in the average levels of the predictors (e.g., BPD patients could, on average, appraise events as more negative). The intercept and slopes were allowed to vary across persons, and were modeled as a function of two diagnostic dummy variables (one for healthy controls and one for the BPD group) at level 2 of the model, leaving out the intercept. As such, the slope for each dummy variable at level 2 reflects the average (reactivity) effect of each appraisal at time *t-1* on affect measured at time t for the healthy controls and for the BPD group, corrected for overlap with the effect of other appraisals. Significant differences between the estimates (i.e., reactivity slopes) of the two binary dummy variables were tested with hypothesis tests for fixed effects using Wald tests. This approach allowed us to model the reactivity effect in response to each appraisal (however, corrected for overlap with other appraisals) in each group separately (i.e., examine whether reactivity occurs in response to an appraisal in each group and examine the direction of that reactivity), and next to compare the strength of the effect between the two groups.

In a second set of analyses, the same models were repeated, this time including all the four BPD specific evaluations. Again, different models were estimated for NA and PA. This analysis approach was chosen to avoid estimating multiple repeated models for each appraisal separately, and to correct for possible overlap between appraisals.

## Results

### Descriptive statistics

Table 1 displays the descriptive statistics for the emotions, the general emotional appraisals, and the BPD-specific evaluations. Regarding the emotions, results showed that on average, the BPD group reported significantly higher levels of NA and lower levels of PA than healthy controls. Regarding the emotional appraisals, the BPD group reported lower emotion-focused coping potential, and lower levels of goal congruence than healthy controls. This means that the situations/stimuli encountered by the BPD patients were on average appraised as more negative, and as being more difficult to cope with emotionally than the situations/stimuli encountered by healthy participants. Regarding BPD-specific evaluations, the BPD group experienced lower levels of trust in self and others, and more disappointment in both self and others, than healthy controls did. However, note that based on these results, we cannot disentangle whether these differences are due to the BPD group experiencing different types of situations, or appraising comparable situations in different ways than healthy controls. To take the differences in these average levels into account in the following analyses, all predictors were centered within-person (see statistical analysis section).

**Table 1:** Descriptive Statistics for the Positive Affect (PA) and Negative Affect (NA) and each of the appraisals under investigation

Emotion	BPD group		Healthy controls		Difference between groups	
	Mean	SE	Mean	SE	*χ*^2^(df)	*p*-value
PA	28.79	2.99	62.61	2.28	80.90 (1)	<.001
NA	36.90	3.87	7.44	1.32	51.97 (1)	<.001
Emotional appraisals						
Goal relevance	59.06	2.80	62.09	3.25	0.50 (1)	>.500
Goal congruence	40.45	3.28	64.75	2.27	37.13 (1)	<.001
Emotion-focused coping potential	45.26	3.16	78.83	2.90	61.20 (1)	<.001
BPD-specific evaluations						
Disappointment in self	44.59	4.29	7.72	1.77	63.10 (1)	<.001
Disappointment in someone else	34.01	4.43	15.32	3.99	9.82 (1)	.002
Trust in self	31.89	3.60	72.66	2.78	80.33 (1)	<.001
Trust in someone else	36.56	4.75	69.24	3.85	28.52 (1)	<.001

*BPD* Borderline personality disorder, *SE* Standard error. Results are based on two-level models with each variable under investigation being predicted by a random intercept at level 1, and diagnostic dummies (leaving out the intercept) at level 2. Differences between diagnostic dummies slopes are tested using general linear hypothesis testing

To examine whether the emotional appraisals and BPD-specific evaluations varied at the moment-to-moment level, thus reflecting states that change over time rather than stable traits, we estimated the amount of variance in each variable at both the moment-to-moment level and the person level. Results showed that a considerable percentage of (total) variance was found at the within-person level (goal congruence: 52%, goal relevance: 62%, emotion focused coping potential: 37%, disappointment in self: 42%, disappointment in others: 46%, trust in self: 33%, trust in others: 29%). This means that, next to variance in scores due to differences between people, a reasonable proportion of the variance for each variable was due to changes within persons over time, also justifying the use of multilevel models.

### Emotional reactivity in NA

We examined whether a similar appraisal or evaluation predicted larger levels of subsequent NA, that is, stronger reactivity in NA (see Table 2) for BPD patients than for the healthy controls. This was done additionally correcting for NA at the previous time point, and correcting for the influence of other appraisals or evaluations. First, we focused on reactivity to general emotional appraisals. We found no significant differences between the BPD group and the healthy controls regarding reactivity to any of the emotional appraisals. This means that both groups responded in similar ways to the emotional appraisals in terms of negative affect.^3^

**Table 2:** Results from multilevel analyses in which Negative Affect (NA) is predicted by a random intercept, by Appraisals and NA at the previous time point at level 1, which are again modeled in function of a Healthy Controls (HC) dummy and a Borderline Personality Disorder (BPD) dummy at level 2

						Difference groups	
Fixed Effect	Coeff.	SE	*t*-ratio	df	*p*-value	χ2 (df)	*p*-value
For intercept, *β*_*0*_							
BPDdummy, *γ*_*01*_	36.58	3.99	9.16	56	< 0.001	48.79 (1)	<.001
HCdummy, *γ*_*02*_	7.31	1.27	5.75	56	< 0.001		
For goal relevance at t-1 slope, *β*_*1*_							
BPDdummy, *γ*_*11*_	0.04	0.03	1.47	56	0.148	1.21 (1)	0.270
HCdummy, *γ*_*12*_	0.01	0.01	0.96	56	0.343		
For goal congruence at t-1 slope, *β*_*2*_							
BPDdummy, *γ*_*21*_	−0.07	0.03	−2.16	56	0.035	0.91 (1)	>.500
HCdummy, *γ*_*22*_	−0.03	0.02	−1.80	56	0.077		
For emotion-focused coping potential at t-1 slope, *β*_*3*_							
BPDdummy, *γ*_*31*_	−0.07	0.03	−2.42	56	0.019	1.73 (1)	0.186
HCdummy, *γ*_*32*_	−0.02	0.03	−0.67	56	0.509		
For NA at t-1 slope, *β*_*4*_							
BPDdummy, *γ*_*41*_	0.34	0.05	7.41	56	< 0.001	0.72 (1)	>.500
HCdummy, *γ*_*42*_	0.28	0.05	5.70	56	< 0.001		
For intercept, *β*_*0*_							
BPDdummy, *γ*_*01*_	36.60	3.99	9.16	56	< 0.001	48.82 (1)	<.001
HCdummy, *γ*_*02*_	7.31	1.27	5.75	56	< 0.001		
For disappointed self t-1 slope, *β*_*1*_							
BPDdummy, *γ*_*11*_	0.05	0.03	1.59	56	0.118	0.02 (1)	>.500
HCdummy, *γ*_*12*_	0.04	0.02	2.30	56	0.025		
For disappointed someone else t-1 slope, *β*_*2*_							
BPDdummy, *γ*_*21*_	0.04	0.01	2.71	56	0.009	7.58 (1)	.006
HCdummy, *γ*_*22*_	−0.01	0.01	−0.91	56	0.366		
For trust self t-1slope, *β*_*3*_							
BPDdummy, *γ*_*31*_	0.04	0.03	1.76	56	0.085	2.09 (1)	0.144
HCdummy, *γ*_*32*_	0.00	0.01	0.26	56	0.796		
For trust someone else t-1 slope, *β*_*4*_							
BPDdummy, *γ*_*41*_	−0.04	0.03	−1.16	56	0.250	0.60 (1)	>.500
HCdummy, *γ*_*42*_	−0.01	0.02	−0.52	56	0.609		
For NA at t-1 slope, *β*_*5*_							
BPDdummy, *γ*_*51*_	0.36	0.04	8.43	56	< 0.001	0.91 (1)	>.500
HCdummy, *γ*_*52*_	0.30	0.05	5.99	56	< 0.001		

*Coeff.* Coefficient, *SE* Standard error. All predictors were entered person-mean centered. Different models were estimated for the general emotional appraisals and BPD-specific evaluations. Differences between diagnostic dummies slopes are tested using general linear hypothesis testing

**Fig. 1:**
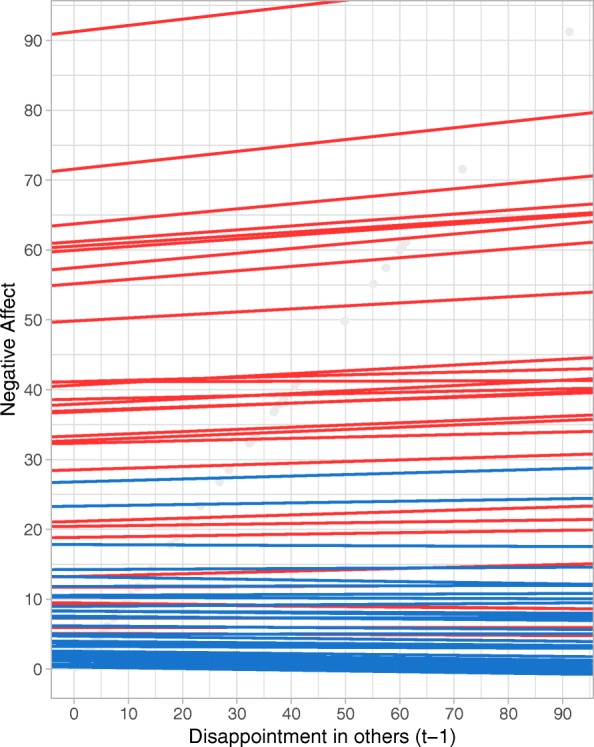
Spaghetti plot visualizing the relationship between disappointment in others and subsequent NA for each person separately, when other BPD related appraisals in the model are set to the average level for each person. Red lines represent persons with BPD, blue lines represent healthy participants

Second, we examined reactivity to the BPD-specific evaluations of self and others. A significant group difference was found only for disappointment in others. Results showed that for the BPD group, more disappointment in someone else was significantly related to higher levels of subsequent NA, indicating strong reactivity. For the healthy controls, no reactivity in response to disappointment in someone else was found.^4^ Figure 1 shows the relation between disappointment in others and subsequent NA for each person, with other BPD related appraisals in the model set to the average level for that person. No significant relationships were found for healthy controls, as indicated by the horizontal blue lines. For persons with BPD (red lines), a positive relationships was found, with largely similar slopes across persons with BPD.

### Emotional reactivity in PA

Next, we examined reactivity in PA (see Table 3). Again, we first focused on levels of PA in response to each of the general emotional appraisals, correcting both for PA at the previous time point and for overlap with the other general appraisals.

**Table 3:** Results from multilevel analyses in which Positive Affect (PA) is predicted by a random intercept, by Appraisals and PA at the previous time point at level 1, again modeled in function of a healthy control (HC) dummy and a Borderline Personality Disorder (BPD) dummy at level 2

						Difference groups	
Fixed Effect	Coeff.	SE	*t*-ratio	df	*p*-value	χ2 (df)	*p*-value
For intercept, *β*_*0*_							
BPDdummy, *γ*_*01*_	29.37	3.21	9.15	56	< 0.001	71.18 (1)	<.001
HCdummy, *γ*_*02*_	62.99	2.36	26.66	56	< 0.001		
For goal relevance at t-1 slope, *β*_*1*_							
BPDdummy, *γ*_*11*_	−0.02	0.03	−0.84	56	0.405	4.32 (1)	.035
HCdummy, *γ*_*12*_	0.05	0.02	2.29	56	0.026		
For goal congruence at t-1 slope, *β*_*2*_							
BPDdummy, *γ*_*21*_	0.06	0.04	1.52	56	0.135	0.06 (1)	>.500
HCdummy, *γ*_*22*_	0.07	0.03	2.19	56	0.033		
For emotion-focused coping potential at t-1 slope, *β*_*3*_							
BPDdummy, *γ*_*31*_	0.07	0.04	1.81	56	0.075	0.86 (1)	>.500
HCdummy, *γ*_*32*_	0.13	0.04	2.93	56	0.005		
For PA at t-1 slope, *β*_*4*_							
BPDdummy, *γ*_*41*_	0.28	0.05	5.65	56	< 0.001	0.19 (1)	>.500
HCdummy, *γ*_*42*_	0.25	0.05	5.54	56	< 0.001		
For intercept, *β*_*0*_							
BPDdummy, *γ*_*01*_	29.39	3.21	9.17	56	< 0.001	71.21 (1)	<.001
HCdummy, *γ*_*02*_	62.99	2.36	26.67	56	< 0.001		
For disappointed self t-1 slope, *β*_*1*_							
BPDdummy, *γ*_*11*_	−0.09	0.04	−2.38	56	0.021	0.00 (1)	>.500
HCdummy, *γ*_*12*_	−0.08	0.03	−2.49	56	0.016		
For disappointed someone else t-1 slope, *β*_*2*_							
BPDdummy, *γ*_*21*_	0.00	0.02	0.13	56	0.894	1.76 (1)	.182
HCdummy, *γ*_*22*_	0.05	0.03	1.78	56	0.081		
For trust self t-1slope, *β*_*3*_							
BPDdummy, *γ*_*31*_	−0.02	0.03	−0.47	56	0.642	0.52 (1)	>.500
HCdummy, *γ*_*32*_	0.01	0.02	0.60	56	0.553		
For trust someone else t-1 slope, *β*_*4*_							
BPDdummy, *γ*_*41*_	0.03	0.04	0.83	56	0.410	3.00 (1)	.079
HCdummy, *γ*_*42*_	0.13	0.05	2.86	56	0.006		
For PA at t-1 slope, *β*_*5*_							
BPDdummy, *γ*_*51*_	0.30	0.04	6.75	56	< 0.001	0.10 (1)	>.500
HCdummy, *γ*_*52*_	0.28	0.05	5.95	56	< 0.001		

*Coeff*. Coefficient, *SE* Standard error. All predictors were entered person-mean centered. Different models were estimated for the general emotional appraisals and BPD-specific evaluations. Differences between diagnostic dummies slopes are tested using general linear hypothesis testing

**Fig. 2:**
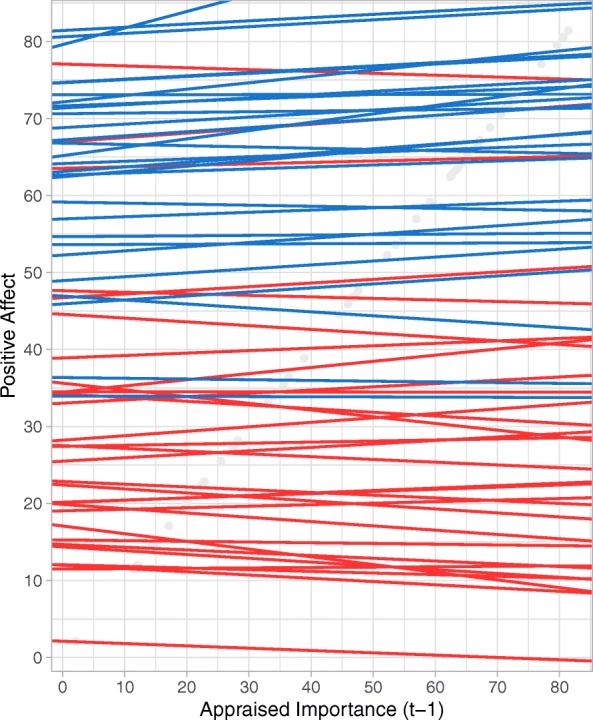
Spaghetti plot visualizing the relationship between the appraised importance on subsequent PA for each person separately, when other general appraisals in the model are set to the average level for each person. Red lines represent persons with BPD, blue lines represent healthy participants

A group difference between patients with BPD and the healthy group was only found for goal relevance. Results indicated a significantly positive effect of the appraised importance on intensity of PA for healthy controls, showing that the more a situation was appraised as important, the higher the subsequent PA for healthy controls. For the BPD group, no reactivity in response to the appraised importance was found for PA, thus showing weaker reactivity compared to the control group.^5^ Figure 2 illustrates the relation between the appraised importance on subsequent PA for each person, with other general appraisals in the model set to the average value for each person. For persons with BPD (red lines), large variability between persons can be seen in the strength and direction of the relationship. Therefore, taken this variability into account, no overall significant association was found for the BPD group. For healthy participants (blue lines), variability between persons was also found, although most persons show a positive relationship.

**Fig. 3:**
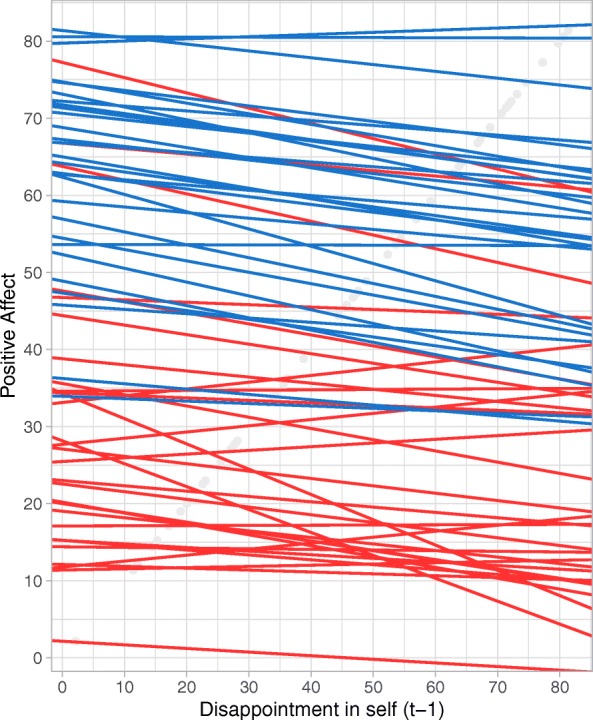
Spaghetti plot visualizing the relationship between disappointment in self and subsequent PA for each person separately, when other BPD related appraisals in the model are set to the average level for each person. Red lines represent persons with BPD, blue lines represent healthy participants

For the BPD specific evaluations, no significant differences were found between the BPD group and healthy controls. However, inspecting effects within the two groups, disappointment in self was significantly and negatively related to subsequent PA for both groups, meaning that high levels of disappointment in self was related to lower levels of subsequent PA in both groups. This effect was not specific to BPD, as no significant differences were found in the magnitude of this effect between the BPD group and the healthy controls.^6^ However, the results show that both groups reacted with a similar decrease in PA in response to more intense experiences of disappointment in self. Figure 3 illustrates the relation between disappointment in self and subsequent PA for each person, with other BPD related appraisals set to average levels for each person. For the healthy control group (blue lines) similar slopes are observed for all persons indicating a negative relationship. For persons with BPD (red lines), more variability can be observed in terms of the strength and direction of the relationship. However, taken together, most persons also show a negative relationship.

## Discussion

The aim of this study was to obtain more insight into the emotion dynamics in daily life of persons suffering from BPD. This was done by examining emotional reactivity to general emotional appraisals (i.e., appraised goal congruence, goal relevance and emotion focused coping potential) and to BPD-specific evaluations (i.e., disappointment and trust in oneself and in others) in daily life of patients with BPD and healthy controls.

In comparison to healthy controls, persons suffering from BPD responded with significantly higher levels of subsequent NA after they experienced more disappointment in someone else. For healthy participants, no significant effect of disappointment in others on subsequent NA was found. These findings suggest that when patients with BPD experience disappointment in others, this triggers strong increases in their negative affect. Regarding reactivity in terms of PA, we found evidence for a weaker reactivity in positive emotions in response to the appraised importance of situations (i.e., goal relevance) for BPD participants compared to healthy participants. More specifically, for healthy participants, the more a situation was appraised as important, the higher the subsequent PA. For the BPD group, such an effect was not found, showing that they were less affectively responsive to the appraised importance of situations in daily life. Additionally, a trigger of emotional reactivity was found in both the BPD group and the healthy controls, where disappointment in self was related to lower levels of subsequent PA. However, the strength of this effect was similar in both groups.

Overall these findings suggest that, not only the occurrence of affect eliciting events, but also appraisals and evaluations of daily life situations, may be important triggers of emotional change over time. In this study, we showed that evaluations of disappointment in others is a potent trigger of emotional changes in those with BPD. On the other hand, we found that BPD patients do not show the same mood-brightening effect in response to the appraised importance of situations that healthy controls do. In fact, the appraised importance had no predictive effect for their consecutive levels of PA, suggesting (at least) partial blunted reactivity in PA.

The finding that those with BPD exhibit stronger reactivity to disappointment in others supports results from a previous daily life study [33] in which it was found that disappointment strongly predicted not only unstable emotions, but also other symptoms such as feeling empty, the experience of intense anger, efforts to avoid abandonment, intense relationships, uncertain sense of self, impulsive behavior, and unreal experiences. This finding is in line with the idea that persons with BPD have impairments related to the maintenance and use of benign mental representations of self and others. They have representations related to fear of others, based on expectations of being disappointed and mistreated by others. These representations are considered important as this disturbed way of thinking about others can drive not only affective instability, but also problematic interpersonal relations and impulsivity [46].

Next, the BPD group exhibited blunted reactivity in PA, in response to the appraised importance of the situation. This effect could be driven by healthy participants experiencing more pleasant events that are appraised as important. However, in our study, we corrected for overlap between the different emotional appraisals, so the increased reactivity for healthy controls in response to the appraised importance, and the absence of this effect for the BPD group is corrected for effects of goal congruence (i.e., how positive or negative something is appraised). It is unclear why healthy controls respond with strong increases in PA based on the appraised importance of situations. In fact, a previous daily life study [15] found no effect of goal relevance on the valence or arousal dimensions of affect in a general student population. However, it does show that PA of persons with BPD is not affected or driven by the importance attributed to situations. Speculatively, it could also suggest that positive emotions of those with more BPD features might be less responsive to the environment, although future studies should further explore this idea and examine reactivity in positive emotions in response to other triggers as well.

These findings partially support Linehan’s biosocial theory of BPD that states that emotion dysregulation of BPD patients includes more intense responses to emotional stimuli [2, 47]. Our study suggests that this might be the case, mainly for negative emotions in daily life, and that mainly disappointment in others is a potent emotional trigger. Moreover, we found indications of a weaker reactivity in positive affect. However, the current findings should also be seen in the light of several null findings obtained in this study. It is remarkable that effects were only found in response to a limited set of factors. Indeed, contrary to our hypotheses, heightened reactivity was only found in response to one of the triggers examined in this study. We hypothesized heightened reactivity in those suffering from BPD, in response to all BPD-related evaluations, and the emotion focused coping potential. These results can be explained by the fact that we corrected for overlap between the different appraisals. Individual appraisals might also contribute to emotional change. However, our analyses showed that when taking overlap between different appraisals into account, especially disappointment in others plays an essential role, above and beyond the effects of other appraisals. Future studies with larger samples sizes should be conducted to further explore the role of the different appraisals, but also to examine the importance of other types of triggers of emotional change. For example, fear of abandonment and instability of interpersonal relationships are shown to be central features of persons suffering from instability [22]. Moreover, regarding evaluations of disappointment and other interpersonal evaluations, the role of different interaction partners (e.g., romantic partner, friends, family etc.) could be crucial. For example, disappointment in a romantic partner could be a more intense trigger of emotional change than disappointment in a colleague. Other representations related to self and others could also be essential triggers of emotional reactivity, since representations related to self-loathing and the fundamental need of care from others, and attributions of others as evil and malevolent are theoretically linked to BPD [46].

Last, our results also indicated that - for both groups - high levels of disappointment in self was related to lower levels of subsequent PA. This shows that (1) persons suffering from BPD exhibit certain emotional reactions that are also typically found in healthy populations, and (2) that disappointment in self is a general strong trigger of emotional change. Indeed, research has indicated that disappointment involves feeling powerless, wanting to get away from the situation, or wanting to do nothing [48], which is related to subsequent worse mood.

A limitation of this study was the use of a small sample. Although our results showed indications for stronger reactivity in NA and a weaker reactivity in PA for the BPD group, more research is needed to replicate our findings with larger samples. However, even with a limited sample, our study was able to reveal the importance of disappointment in others for emotional change, indicating the relative importance of this trigger. Second, our sample consisted of inpatients that were mostly female. Although daily life in a psychiatric hospital can still be very emotionally challenging, it is not clear whether similar patterns of results would be found for patients in their own typical environment. Moreover, it is unclear whether gender would affect this pattern of responding. Third, an important limitation is the lack of standardized clinical interview to confirm the formal diagnosis of BPD and to assess the absence of psychopathology in the control group. Still, patients were recruited in BPD specialized treatment units, received a diagnosis of BPD as part of the intake procedure in the unit, and scored very high on a self-report measure of BPD symptomatology. Moreover, healthy participants were screened for (past) psychopathology using self-report questionnaires, and scored very low on questionnaires assessing BPD and depression, thus showing no indications of psychopathology. Still, replications with other samples that were carefully assessed with standardized clinical interviews are needed in the future. Fourth, the compliance to the experience sampling protocol was modest (65%) for the patient group. However, most participants had a compliance of at least 50%, resulting in 40 repeated assessments per person. Moreover, follow-up analyses indicated that results were largely similar if we corrected for differences in compliance between the groups. Fifth, due to the sampling frequency, the average time interval between consecutive measurements was 1.33 h, which implies that we examined emotional reactions to triggers that, on average, appeared 1.33 h earlier. It is not clear whether the patterns of results would be different for a different time interval.

Our findings may have implications for clinical practice. This study highlights the importance of appraisal processes, next to affect eliciting events, for understanding why emotional states in persons with BPD tend to abruptly change from one moment to the next. Emotion regulation skills training, often offered to these individuals as part of their treatment should therefore not only focus on modulating emotional responses to a variety of typical affects eliciting events, but also make people aware of appraisal processes that take place, their impact, and teach patients how to effectively deal with them, using cognitive interventions. This proposition is in line with approaches used during dialectical behavioral therapy (DBT [2]). DBT includes emotion regulation skills training, distress tolerance training and interpersonal skills training, during which appraisal processes are also tackled in order to improve emotional and interpersonal functioning. These approaches are supported by our findings.

## Conclusion

To conclude, this study presents preliminary evidence that appraisal processes in daily life might be important for understanding why emotions abruptly change in persons with BPD. In this study, we showed that BPD patients exhibit increased emotional reactivity in daily life in their negative emotions in response to disappointment in others. Moreover, weaker reactivity was found in positive affect in response to the appraised importance of a situation. These findings provide more insight into altered emotional reactivity as a potential process underlying emotional instability in the daily life of BPD patients.

## Abbreviations

ADP-IV: The Assessment of DSM-IV personality disorders
BPD: Borderline personality disorder
ESM: Experience sampling method
HC: Healthy controls
NA: Negative affect
PA: Positive affect
PDSQ-MDD: Psychiatric diagnostic screening questionnaire—major depressive disorder

## Footnotes

1 Note that the data described in this paper is part of a larger ESM project. Based on this project, previous papers on the occurrence of switches between opposite-valenced emotional states [4] and the relationship between non-suicidal self-injury and emotional states [37, 38] have been published, but not on appraisals and their relationship to emotional states.

2 The original sample consisted of 34 patients. However, four were excluded due to a poor compliance to the ESM protocol (compliance < 25%).

3 If we corrected for differences in compliance, by adding compliance grand-mean centered at level 2, still no significant effects were found.

4 After correction for differences in compliance between groups, we still found a significant positive effect of disappointment in someone else on subsequent NA in the patient group (estimate = .04, SE = .01, *p* = .007) that was significantly higher (*p* < .001) than for healthy controls (estimate = −.03, SE = .01, *p* = .073).

5 After correction for differences in compliance between groups, we found that the difference between the two groups in terms of reactivity to the appraised importance became smaller, and now only marginally significant (*p* = .077).

6 If we corrected for differences in compliance between groups in models regarding reactivity in PA in response to BPD-specific evaluations, we still found a negative effect of disappointed in self on subsequent PA for both groups (HC: estimate = −.13, SE = .04, *p* < .001; patient: estimate −.07, SE = 0.03, *p* = .033) that did not differ between groups (*p* = .200). However, we also found a positive effect of disappointment in others on subsequent PA for HCs (estimate = .08, se = .03; *p* = .011) but not for the patient group (estimate = .00, se = .02, *p* = .846). This effect differed significantly between groups (*p* = .019).
